# Interphase Influence on the Effective Thermal Conductivity Coefficients of Fiber Composites

**DOI:** 10.3390/ma18010101

**Published:** 2024-12-30

**Authors:** Jan Turant

**Affiliations:** Department of Mechanical Engineering, Informatics and Chemistry of Polymer Materials, Faculty of Material Technologies and Textile Design, Lodz University of Technology, Zeromskiego 116, 90-924 Lodz, Poland; jan.turant@p.lodz.pl

**Keywords:** effective thermal conductivity, composite materials, interphase, finite element methods

## Abstract

This study proposes a two-scale approach to determining the effective thermal conductivity of fibrous composite materials. The analysis was first carried out at the fiber–interphase level to calculate the effective thermal conductivity of this system, and next at the whole composite structure level. At both scales, the system behavior was analyzed using the finite element method. To determine the effective thermal conductivity for the fiber–interphase system, an inverse problem was solved, while a simple unidirectional heat conduction test was performed for the entire composite. The simulations were carried out for typical fibrous composites: carbon fibers–epoxy resin and glass fibers–epoxy resin. The results showed a significant impact of realistically observed interphase thicknesses on the heat conduction properties of the tested composites.

## 1. Introduction

The presence of an interphase between a composite matrix and its reinforcement is often an overlooked factor influencing the thermomechanical properties of composites, even though its impact can be significant. The characteristics of the geometric and material properties of the interphase are challenging due to its small size, which can nevertheless vary significantly [1]. The physical characteristics of the interphase are described in numerous studies [2,3,4]. The authors emphasize the importance of physical and chemical factors influencing the characteristics of the interphase. Key factors include the technological aspects of composite material production and the impact of various chemical agents that enhance the physical and functional properties of the composite. Coupling agents can be distinguished among additives with the greatest impact on the size and properties of the interphase, enhancing adhesion between the composite filler and the matrix [5]. These additives significantly influence the transition zone from the fiber material to the matrix material. These agents are among the many used in the production processes of composite materials. Among the commonly used agents there are those responsible for forming protective films that safeguard fibers from damage during composite manufacturing, lubricants that coat the fibers to ensure smooth passage through delivery equipment, surfactants and emulsifiers that moisturize fiber surfaces, antistatic agents applied to fibers, and antioxidants [2]. Consequently, multiple layers of additives may be formed on raw fibers, which must bond tightly with the composite matrix. The structure of the interphase is also influenced by the curing rate of the matrix [6] as well as the surface irregularities of the fiber.

The thickness of the interphase is one of its most critical parameters. Numerous studies have focused on measuring the interphase thickness using various methods. A comprehensive review of these studies is presented in [1]. The article provides interphase thickness values for different fiber–matrix combinations. Additionally, it includes an analysis of interphase thickness based on phase imaging atomic force microscopy and nanoindentation techniques. The interphase thicknesses identified in this study are in the range of 2–3 µm for glass fibers in an epoxy matrix. These observations align with findings from other researchers studying this type of composite. Based on the publications analyzed by this author, the interphase thicknesses for carbon fiber–epoxy resin composites range from 1 to 8 µm. Smaller interphase thicknesses than those cited above were obtained in studies demonstrated in [7] and [8], measuring 0.5 µm and 0.9 µm, respectively. In [7], a micromechanical approach was employed, while [8] utilized atomic force microscopy phase imaging and nanoindentation techniques. The interphase thicknesses observed for composites based on carbon fibers and epoxy resin are smaller, ranging between 0.003 µm and 0.5 µm [1]. In the article by [6], the interphase thicknesses for such composites were investigated, revealing significant differences depending on the curing speed of the epoxy resin—0.02 µm for a fast-curing matrix and 0.04 µm for conventional curing rates. The interphase thickness was calculated here by taking a scanning line from the fiber to the matrix radially in the modulus image. In [9], the interphase thicknesses for various connections of carbon fiber and resins were investigated, resulting in a measured thickness of approximately 0.05 µm.

Due to the high significance of the mechanical parameters of composites, the literature on the influence of the interphase on the mechanical properties of composite materials is quite extensive. Examples of studies addressing this topic can be found in [5,6,7,9,10].

Studies on thermal conductivity coefficients for composite materials represent another important area of research on the properties of these materials. The diversity of research directions in this field is considerable. Authors seek effective thermal conductivity coefficients for practically relevant structures, assuming random distributions of the filler [11,12,13], or analyze thermal conductivity of new practically significant materials [14,15,16,17]. However, in the vast majority of works, the impact of the interphase is not considered.

The analysis of the impact of interphases on thermal conductivity coefficients is a rarely discussed topic, despite its potentially significant importance. Among the few articles available, some assume an effective thermal conductivity coefficient for the interphase, while others attempt to determine this property. The first group of articles includes [18,19,20], while the second group includes, for example, ref. [21]. In [18], the impact of interphase thermal conductivity on the properties of composites with various filler types, such as disks, cylinders, and spheres, was considered. The calculations were performed for a specified interphase thickness and an assumed thermal conductivity coefficient “close” to that of the composite matrix. The calculations were carried out using the finite element method. In [19], a new finite element (coating/fiber element) was proposed, where the interphase thermal conductivity was assumed to be the arithmetic mean of the thermal conductivities of the matrix material and the fiber. In [20], the effective thermal conductivities for composites with imperfect matrix–fiber bonding through an interphase were investigated. The studies were carried out for regular fiber arrangements in square and hexagonal patterns. The effective thermal conductivity was again assumed to be the arithmetic mean of the corresponding thermal conductivities of the matrix and the fiber. In [21], theoretical considerations were presented regarding the effective thermal conductivity for a composite where the interphase surface shape oscillates around a curved surface. The author determined the effective thermal conductivity for the oscillating transition zone and, in the next step of homogenization, the impact of the homogenized interphase on the effective thermal conductivity was taken into account. The approach presented here only takes into account an idealized contact surface shape, without accounting for other factors influencing the size and properties of the interphase.

The presented article examines the effect of the interphase on the effective thermal conductivity coefficients of composite materials filled with long, parallel-aligned fibers. A two-scale/two-step process for determining the effective thermal conductivity coefficients is proposed. The calculations were performed for typical fiber-reinforced composites consisting of carbon or glass fibers and an epoxy matrix. During the numerical experiments, the impact of the interphase thickness and the fiber volume fraction of the composite matrix were investigated. The finite element method was used at both stages of the analysis.

## 2. Problem-Solving Methodology

**Figure 1:**
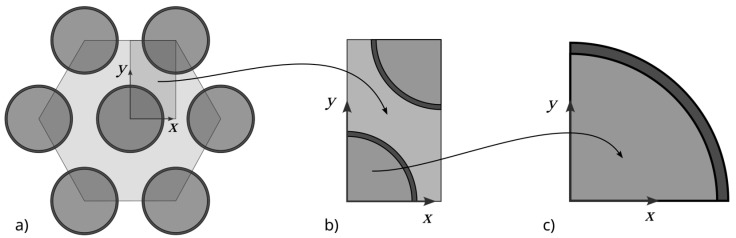
(**a**) Hexagonal fiber arrangement structure, (**b**) representative volume element, and (**c**) 1/4 of the fiber cross-section together with the interphase.

Determining the influence of the interphase on the effective thermal conductivity coefficient of composite materials filled with long fibers arranged in parallel within the matrix was carried out for a hexagonal fiber arrangement (Figure 1a). Such an arrangement of fibers provides the highest potential fiber volume fraction of the composite, reaching a value of (πr^2^/2)/(3^0.5^r^2^) ≅ 0.907 (the maximum ratio of the area occupied by a fiber with radius r to the total area of the RVE). The analysis of such a structure can be carried out by examining the RVE (representative volume element). In the case of a hexagonal structure, this element can be represented by a rectangle containing fiber quarters, as shown in Figure 1b. The fiber elements in this structure are surrounded by an interphase of assumed constant thickness (Figure 1c).

To determine the effective thermal conductivity coefficients, methods based on steady-state heat flow were utilized. Such a problem is described by the heat equation and Fourier’s law [22]:

(1) divq+f=0, q=−k∇T ,

where **q** and *f* denote a heat flux intensity and a heat source respectively, **k** is the matrix of thermal conductivity coefficients, and ∇*T* denotes the gradient of the temperature field. To be able to solve this type of problem, it is necessary to determine proper boundary conditions, which will be presented while describing particular problems.

### 2.1. Two Stages of Analysis

To identify the influence of the interphase on the composite’s effective thermal conductivity coefficient, the analysis was carried out in two stages. In the first stage, the effective thermal conductivity coefficient was determined for the fiber with the interphase, and in the second stage, for the composite’s representative volume element (RVE) in the direction of the y-axis, the assumed transverse direction of heat transfer for the composite (Figure 1), was determined.

This approach makes the calculations independently of the specific fiber distribution within the composite matrix. Consequently, the effective thermal conductivity coefficients for a fiber–interphase system determined in this way can be used for studying other fiber distributions in a matrix than the one assumed in this work. After completing the first stage, the fiber and interphase body are treated as homogeneous. As a result, in the second step of the analysis, the composite consists of only two phases instead of three.

#### 2.1.1. First Stage of Analysis

**Figure 2:**
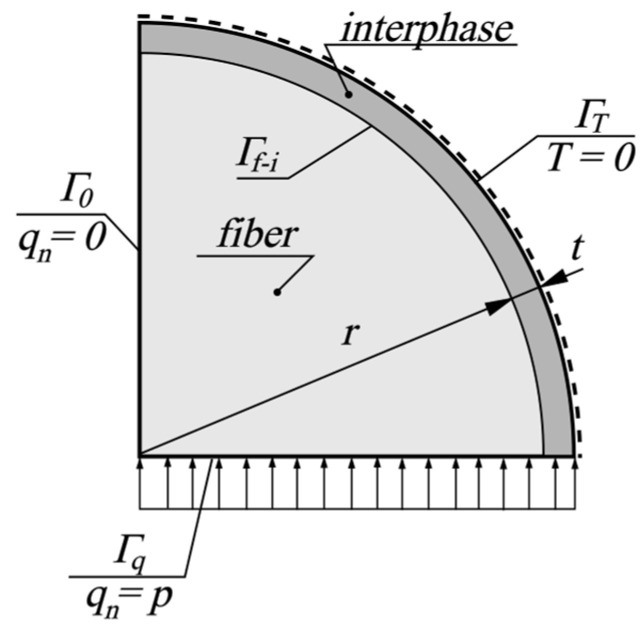
Schema of fiber–interphase system.

In the first stage of solving the problem, the thermal behavior of the fiber and interphase cross-section is considered. Due to its symmetry, one-fourth of the cross-section is considered. The schema of the problem is shown in Figure 2.

In Figure 2, *r* denotes the radius of the fiber cross-section, and *t* is the thickness of the interphase, while the boundary conditions are defined as follows:

(2) $$T=0\;on\;\Gamma_{T},\;q_{n}=q\cdot n=0\;on\;\Gamma_{0},\;q_{n}=q\cdot n=p\;on\;\Gamma_{q}\;.$$

where *T* denotes temperature, *q_n_* is the normal heat flux, *p* is its prescribed value at the boundary Γ*_q_*, and **n** is the normal unit vector of the boundary line at a chosen point. At the boundary Γ*_f-i_* (the interface between the fiber and the interphase), continuity of the temperature fields is assumed.

Determining the effective thermal conductivity coefficient for the fiber–interphase system requires an identification process for the homogenized properties of the fiber–interphase system. To realize this inverse problem, an analysis of the fiber behavior, as shown in Figure 2, must be carried out. Based on the analysis, the temperature distribution on the boundaries of the specimen with unknown a priori temperatures can be obtained (thermally insulated and loaded boundaries).

In the identification process, the obtained temperature distribution must be compared with the distributions obtained for a specimen made from homogeneous material. As a measure of the correlation between the behavior of the specimen with a heterogeneous structure (fiber–interphase) and the homogenized specimen with a prescribed thermal conductivity coefficient, a function was chosen that represents the Eulerian measure of the distance between the solutions for the non-homogeneous and homogeneous systems:

(3) $$F(k_{a})=\sqrt{{\sum_{i=1}^{n}\left(T_{nh}^{i}-T_{h}^{i}\right)}^{2}}$$

The symbols $T_{nh}^{i}\;\mathrm{and}\;T_{h}^{i}$ denote here the temperature at the *i*-th boundary point for the non-homogenized and homogenized materials, respectively, *m* is the number of points on the tested boundaries, and *k_a_* is the assumed thermal conductivity coefficient for the homogenized fiber–interphase system. The effective thermal conductivity coefficient of the fiber–interphase system must be determined by solving the minimization problem:

(4) $$\min F(k_{a})\to \;k_{fi}$$

where *k_fi_* is the effective thermal conductivity coefficient for the fiber–interphase system.

#### 2.1.2. Second Stage of Analysis

**Figure 3:**
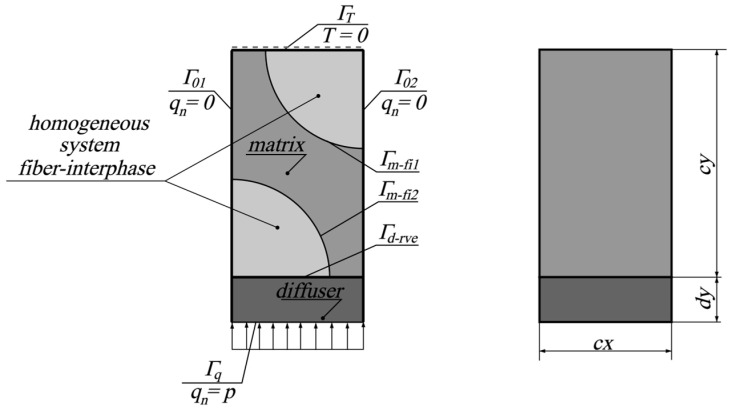
RVE specimen schema.

In the second stage, the effective thermal conductivity coefficient for the RVE is determined. To achieve this, a virtual test of unidirectional heat transfer through the RVE can be carried out. The schema of the problem being solved at this stage is shown in Figure 3.

In the schema shown in Figure 3, an additional heat dispersing material is visible (diffuser), which aims to equalize the temperature at the boundary Γ*_d-rve_* of the RVE, simplifying the calculation of the effective thermal conductivity coefficient, according to the methodology presented in [16]. The shown specimen is thermally insulated along the boundaries Γ_01_ and Γ_02_, subjected to a heat flux with intensity *p* on the boundary Γ*_q_*, and subjected to a prescribed temperature of 0 °C at the boundary Γ*_T_*. Consequently, the boundary conditions can be written as follows:

(5) $$T=0\;on\;\Gamma_{T},\;q_{n}=q\cdot n=0\;on\;\Gamma_{01}\cup \;\Gamma_{02},\;q_{n}=q\cdot n=p\;on\;\Gamma_{q}\;$$

On the boundaries where different materials are in contact (Γ*_d-rve_*, Γ*_m-fi1_*, and Γ*_m-fi2_*), the continuity of the temperature fields is assumed. By calculating the temperature *T*_0_ at the boundary Γ*_d-rve_*, the effective thermal conductivity coefficient can be determined using the following formula:

(6) $$k_{ye}=\frac{cy}{T_{0}}p$$

## 3. Methods

In both stages of the analysis, the finite element method was used. All necessary procedures and functions were written in the FORTRAN programming language (GNU FORTRAN Compiler 6.3.0-1). The implemented program performs multiple finite element analyses, solving the primary problem of the first stage (reference solution), auxiliary problems during the identification of the fiber–interphase system properties, and finally the problem of determining the effective thermal conductivity coefficient of the composite (RVE properties). The program also provides the option to conduct only the first step of the two-stage analysis described in earlier sections, returning solely the calculated effective thermal conductivity coefficients for the fiber–interphase system.

The discretization of the fiber, interphase, and composite matrix areas was carried out in a way that suited the specifics of the problem being solved. The discretization was performed using three-node triangular elements. The discretization process was carried out automatically, depending on the fiber cross-sectional diameter and the interphase thickness. The automation of this process allowed for the automation of studies on the selected range of interphase thickness variations and fiber volume fractions in the composite matrix, eliminating the need for tedious manual adjustment of parameters for the tested specimens.

**Figure 4:**
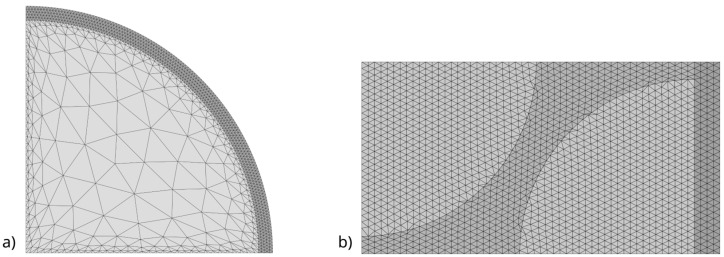
(**a**) Discretization of fiber with interphase and (**b**) discretization of RVE along with diffuser.

An example of the chosen discretization method for the fiber cross-section with the interphase is shown in Figure 4a, while Figure 4b illustrates an example of the RVE discretization along with the diffuser material area. The automatic discretization procedure for the fiber and interphase region was programmed specifically for the analysis conducted. The discretization procedure for the RVE was implemented in a manner analogous to that presented in [23].

As a result of the analyses carried out in the first stage (homogenization of the fiber and interphase), the temperatures on the boundaries with the prescribed heat flux for the heterogeneous structure (composed of fiber and interphase) were determined. This distribution was treated as the reference distribution for the process of determining the effective thermal conductivity coefficient of the fiber–interphase system. In this process, the thermal conductivity coefficient for a specimen with the same external shape as the reference specimen and made of a homogeneous isotropic material was determined. It was assumed that the temperature distributions on the boundaries of the reference specimen and the specimen under investigation, made of a homogeneous material, should be the same. For the analysis of the behavior of the structure with homogeneous properties, the same finite element method mesh was used as for the reference structure made of two materials—the fiber and the interphase. For the purpose of finding the properties of the specimen that behaves like the reference structure, a minimization process was carried out, described by Equations (3) and (4).

Due to the existence of a single design variable (*k_a_*) in problem (4), the quadratic interpolation method was used. The starting point for the iterative process of this method was chosen as the thermal conductivity coefficient of the fiber material, expecting that the effective thermal conductivity coefficient of the system would not drastically differ from it.

As a result of the analyses carried out in the second stage, the temperature at the RVE–diffuser boundary was determined, and the effective thermal conductivity coefficient for the entire composite was calculated using Formula (6).

## 4. Results

To achieve the objectives set out in the article, calculations were carried out for two commonly used types of fiber-reinforced composites: epoxy resin–carbon fibers and epoxy resin–glass fibers.

The properties of filler fibers, even within the same base material, can vary significantly due to differences in manufacturing technologies and parameters. For instance, the thermal conductivity of the most commonly used carbon fibers can range from 9 to 160 W/(m K) [24], while for glass fibers, it ranges from 0.55 to 1.4 W/(m K) [25]. The diameter of typical carbon fibers varies between 5 and 11 µm [24] and, for glass fibers, it ranges from 5 to 24 µm [2]. The thermal conductivity of epoxy resins can range from 0.15 to 0.25 W/(m K) [26]. The values used for the calculations are shown in Table 1.

Numerical tests will be carried out for the systems being combinations of components **1**-**5**, **2**-**5**, **3**-**5**, **4**-**5** shown in Table 1.

As outlined in the introduction of this article, the geometric and material properties of the interphase connecting the fibers to the matrix depend on numerous physical and chemical factors. The thickness of the interphase has been investigated in many studies mentioned in the introduction. The interphase thickness can range from as little as 0.003 µm to as much as 8 µm [1]. Investigating the properties of the interphase is challenging and, consequently, it is typically assumed that the interphase possesses average thermal conductivity due to the “interpenetration” of the fiber and resin materials.

In the numerical tests, variations in interphase thickness were analyzed, ranging from zero to the maximum (typical) thicknesses observed in studies [1]. For carbon fibers, a maximum interphase thickness of 0.5 µm was assumed, while for glass fibers, it was set at 3 µm. The analysis of the effective thermal conductivity coefficients for composite materials was carried out for various fiber volume fractions in the composite matrix.

**Figure 5:**
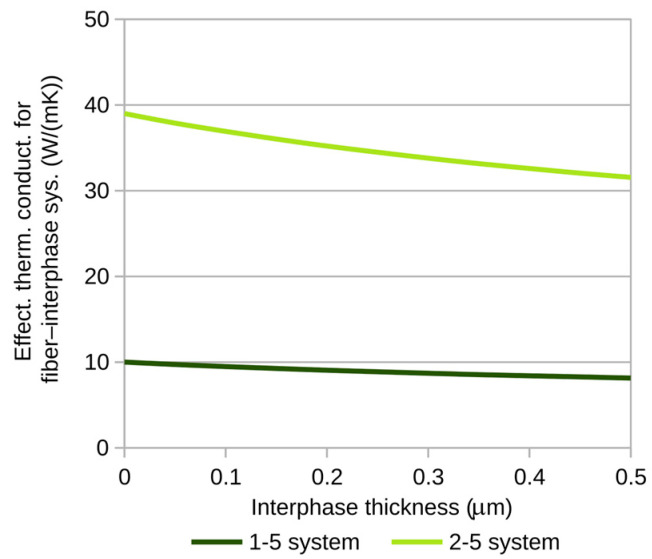
Effective thermal conductivity of fiber–interphase systems containing carbon fibers.

**Figure 6:**
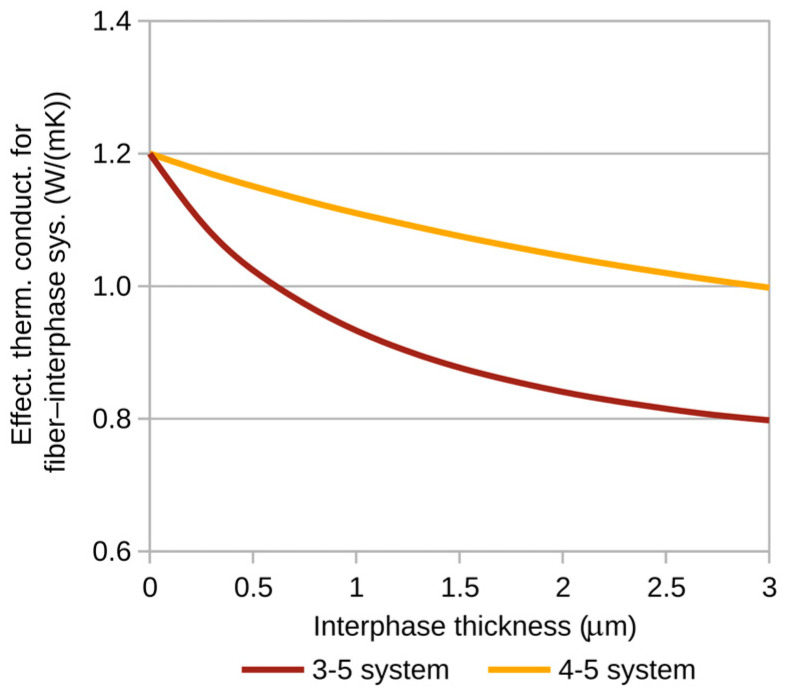
Effective thermal conductivity of fiber–interphase systems containing glass fibers.

Figure 5 and Figure 6 show the variations in the effective thermal conductivity coefficient of the fiber–interphase system for the previously proposed fiber–matrix combinations. The calculations were performed for a heat flux of *p* = 10^−3^ W/m (Figure 4).

The maximum relative differences (with respect to the fiber material) between effective thermal conductivity coefficients for the analyzed fiber–matrix combinations are presented in Table 2.

The large maximum differences in effective thermal conductivity coefficients for carbon fibers are mostly a function of the substantial disparity in thermal conductivity between the fiber material and the matrix. For glass fibers, the observed differences are more influenced by the significant contribution of the interphase. It is important to note that the observed changes in the thermal conductivity coefficient for the fiber–interphase system also entail a change in the effective fiber diameter, which includes the interphase thickness as well. The effective thermal conductivity coefficients calculated here can be used to determine effective coefficients for any fiber distribution within the composite matrix.

**Figure 7:**
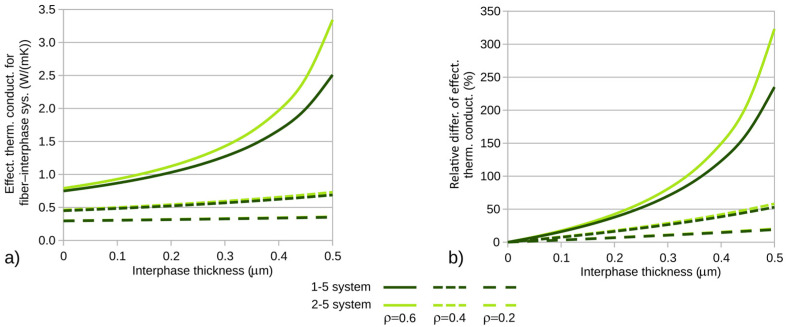
**(a**) Effective thermal conductivity and (**b**) relative differences in thermal conductivity for composites containing carbon fibers.

**Figure 8:**
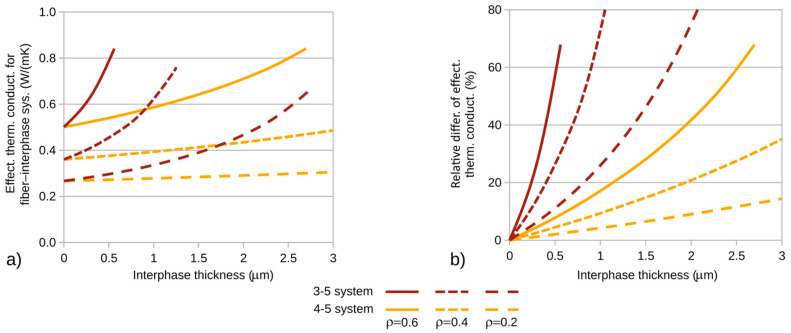
(**a**) Effective thermal conductivity and (**b**) relative differences in thermal conductivity for composites containing glass fibers.

In the studies conducted, the effective coefficients for the fiber–interphase system were calculated to determine the impact of the interphase on the thermal conductivity coefficient of composites with a hexagonal fiber arrangement (Figure 1). The calculations were performed for a heat flux of *p* = 10^−3^ W/m (Figure 5) and the RVE dimensions were determined by the considered fiber volume fraction of the composite and the fiber diameter. Figure 7a and Figure 8a present the calculated effective thermal conductivity coefficients of composites for the fiber–matrix systems analyzed in the previous step.

To better observe the interphase’s influence on the composite’s thermal conductivity coefficient, graphs of the relative differences of the investigated coefficients (relative to the coefficients of composites with zero interphase) were created. The corresponding differences are shown in Figure 7b and Figure 8b. The calculations were performed for variable fiber volume fractions of the composite. In each of the plots (Figure 7 and Figure 8), the corresponding quantities are illustrated as a function of interphase thickness for three selected fiber volume fractions of the composite: ρ = 0.2, 0.4, and 0.6.

It is noteworthy that the fiber–interphase volume fraction of the composite system is always greater than the theoretical volume fraction. For all analyzed cases, computations were carried out up to the specified interphase thickness or until contact occurred between fiber–interphase systems within the RVE. The effect of termination of calculations due to the filling of the RVE cell with the effective material of the fiber–interphase system is visible for systems 3-5 and 4-5 (Figure 8)—systems containing glass fibers and possessing relatively large maximum interphase thicknesses.

The maximum relative differences in the computed effective thermal conductivity coefficients are shown in Table 3.

Significant increases in the effective thermal conductivity coefficients are clearly observed with the increase in density ρ. This effect is caused by the quadratic influence of the effective radius of the fiber–interphase system on the effective fiber volume fraction of the composite. In the extreme cases considered, the computed thermal conductivity coefficient was more than two and three times greater than that calculated for a composite material with zero interphase thickness.

**Figure 9:**
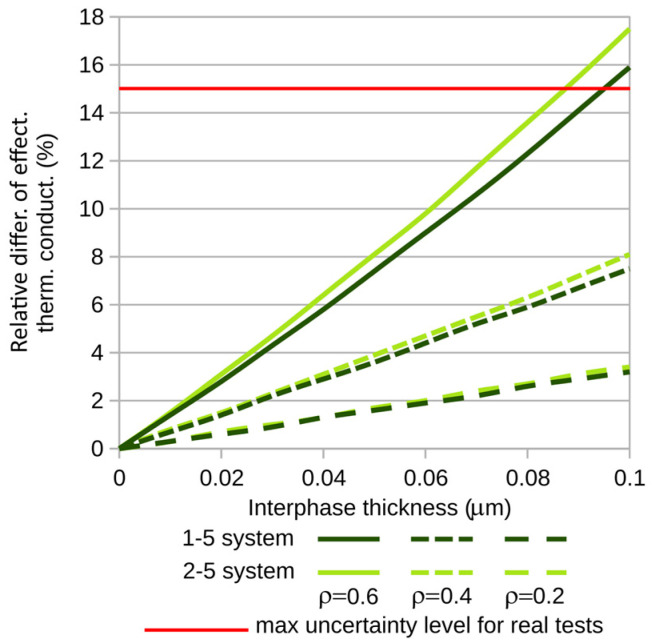
Relative differences of thermal conductivity for composites containing carbon fibers.

**Figure 10:**
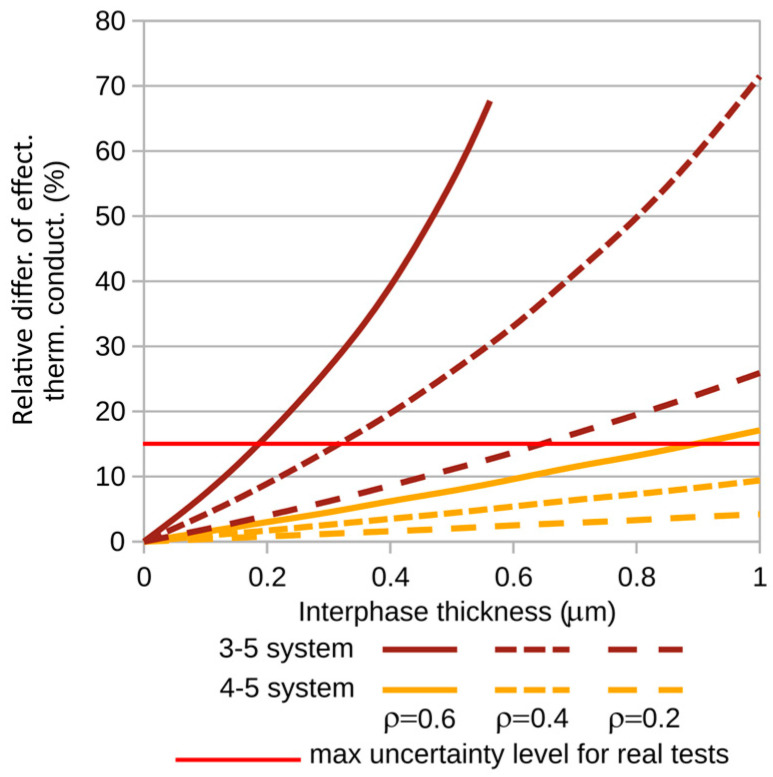
Relative differences in thermal conductivity for composites containing glass fibers.

To further illustrate the analyzed problem, Figure 9 and Figure 10 show the relative differences in thermal conductivity coefficients for small, practically observed ranges of interphase thickness variations. For carbon fibers, the maximum interphase thickness considered was 0.1 µm, while for glass fibers it was 1 [µm]. The red line indicates the maximum uncertainty level in thermal conductivity measurements typically encountered in real-world experiments [27]. This level is shown here to highlight the range of variations in thermal conductivity which are too large—too significant to neglect the influence of the interphase on the studied phenomenon.

## 5. Discussion

The presented results indicate the potential for a significant impact of interphase thickness on the thermal conductivity of composite materials. For carbon and glass fibers, which are typically used as reinforcement in composites, the increase in thermal conductivity coefficients is a result of the distinct material and geometric parameters of the fibers and the matrix.

For carbon fibers, the key parameter is their relatively high thermal conductivity (9–160 W/(m K)) compared to the thermal conductivity of epoxy matrices (0.15–0.25 W/(m K)). In the case of glass fibers, the observed larger interphase thicknesses (reaching dimensions comparable to the fiber diameter) play a more critical role, while their thermal conductivity (approximately 1 W/(m K)) is much closer to the thermal conductivity of polymer resins. The influence of interphase thickness is particularly noted for high fiber volume fractions of the composites.

The study was carried out for potentially large, realistically occurring interphase thicknesses. The observed significant differences in thermal conductivity coefficients (in comparison to composites with zero interphase thickness) may not be common. However, it is worth noting that the analysis of the effective thermal conductivity behavior for relatively small, more frequently encountered interphase thicknesses highlights the necessity of considering the interphase in simulation calculations. This observation is especially important for higher fiber volume fractions of the composite and for glass fibers with small diameters.

## 6. Conclusions

The study employed computational simulation techniques based on the finite element method during the analysis phase. For solving the inverse problem, which determined the effective thermal conductivity of the fiber–interphase system, the quadratic interpolation method was used. A two-scale simulation analysis of the interphase’s impact on the behavior of fiber-reinforced composites was proposed in the study. The analysis aimed to demonstrate the influence of the interphase on the effective thermal conductivity of composite materials. The calculations revealed a significant impact of the interphase thickness on the effective thermal conductivity coefficients, even for cases with small interphase thicknesses.

The proposed approach enables the use of effective thermal conductivity coefficients of the fiber–interphase system for any fiber arrangement within the composite. Moreover, it reduces the analysis to a problem involving only two phases: the phase of the effective fiber material and the matrix. This simplification of the computational model facilitates the discretization of the matrix and effective fibers domains, making it independent of the internal structure of the fiber–interphase system.

In the study, it was necessary to assume a thermal conductivity coefficient for the interphase. Due to the lack of data in the literature on the magnitude of such a coefficient, it was assumed—consistent with a common approach in the literature—that this coefficient is the arithmetic average of the thermal conductivity coefficients of the fiber material and the matrix. This assumption stems from the gradual transition of the fiber material (with a rough surface) into the matrix material. Such an assumption appears to be valid only for very small interphase thicknesses. In practice, the thickness of the interphase is determined not only by the properties of the fiber but primarily by the technological conditions of the composite manufacturing process and the chemical agents introduced during production. Agents that enhance adhesion between the fiber and the matrix are particularly significant in this regard. Due to the diversity of technologies and chemical agents used, it is challenging to precisely define the interphase structure, making simulation studies of such structures very difficult. Simulation-based investigations could potentially be implemented during the assessment of thermal properties under strictly defined composite production conditions. This could enable a better definition of the structure and the physico-chemical parameters of the interphase. Given the significant uncertainty connected with interphase parameters, methods based on granular computing and fuzzy numbers [28] appear particularly promising and could be interesting as a field of further investigation. These methods could be applied during the process of determining interphase parameters.

From the perspective of accurately defining the thermal conductivity of fibrous composite materials, minimizing the thickness of the interphase is critical. The obtained simulation results suggest the need for strict maintaining of technological processes to achieve minimal interphase thicknesses. From the perspective of thermal properties, a rigorous approach to the production process is particularly crucial for composites reinforced with glass fibers.

## Figures and Tables

**Table 1:** Assumed material and geometrical properties of fibers and resin matrix.

No	Components	Conductive Coefficient [W/(m K)]	Diameter [µm]
1	Carbon fiber	10	5
2	Carbon fiber	39	5
3	Glass fiber	1.2	5
4	Glass fiber	1.2	24
5	Epoxy risen	0.2	N/A

**Table 2:** Maximum relative differences.

Fiber–Matrix Combination	Maximum Relative Difference λ*_f-i_* [%]
1-5	19
2-5	19
3-5	34
4-5	17

**Table 3:** Maximum relative differences in thermal conductivity coefficients for the considered composites.

Fiber–Matrix Combination	Maximum Relative Difference λ*_e_* [%] for Various Fiber Volume Fractions of the Composite		
	ρ = 0.2	ρ = 0.4	ρ = 0.6
1-5	19	53	235
2-5	20	58	323
3-5	153	109	68
4-5	14	35	68

The highlighted cells of Table 3 contain the values of the effective thermal conductivity coefficients for cases where the maximum assumed thickness of the interphase was not reached—indicating earlier filling of the RVE by the fiber–interphase system.

## Data Availability

The original contributions presented in this study are included in the article/Supplementary Material. Further inquiries can be directed to the corresponding author.

## Supplementary Materials

The following supporting information can be downloaded at https://www.mdpi.com/article/10.3390/ma18010101/s1.
